# Hyberbaric oxygen as sole treatment for severe radiation - induced haemorrhagic cystitis

**DOI:** 10.1590/S1677-5538.IBJU.2016.0451

**Published:** 2017

**Authors:** Athanasios Dellis, Athanasios Papatsoris, Vasileios Kalentzos, Charalambos Deliveliotis, Andreas Skolarikos

**Affiliations:** 1 2nd Department of Surgery, National and Kapodistrian University of Athens,Aretaieion Academic Hospital, Greece;; 2 2nd Department of Urology, University of Athens,Sismanoglio General Hospital, Athens, Greece;; 3 Department of Diving and Hyperbaric Oxygen, Naval and Veterans Hospital, Athens, Greece

**Keywords:** Urinary Bladder, Cystitis, Hematuria, Oxygen Inhalation Therapy

## Abstract

**Purpose:**

To examine the safety and efficacy of hyperbaric oxygen as the primary and sole treatment for severe radiation-induced haemorrhagic cystitis.

**Materials and methods:**

Hyperbaric oxygen was prospectively applied as primary treatment in 38 patients with severe radiation cystitis. Our primary endpoint was the incidence of complete and partial response to treatment, while the secondary endpoints included the duration of response, the correlation of treatment success-rate to the interval between the onset of haematuria and initiation of therapy, blood transfusion need and total radiation dose, the number of sessions to success, the avoidance of surgery and the overall survival.

**Results:**

All patients completed therapy without complications with a mean follow-up of 29.33 months. Median number of sessions needed was 33. Complete and partial response rate was 86.8% and 13.2%, respectively. All 33 patients with complete response received therapy within 6 months of the haematuria onset. One patient needed cystectomy, while 33 patients were alive at the end of follow-up.

**Conclusions:**

Our study suggests the early primary use of hyperbaric oxygen for radiation-induced severe cystitis as an effective and safe treatment option.

## INTRODUCTION

The use of hyperbaric oxygen (HBO) as a treatment modality of radiation-induced lesions is not new, while about a decade ago the European Society for Therapeutic Radiology and Oncology and the European Committee for Hyberbaric Medicine underlined the indications of HBO in the treatment of radio-induced manifestations in normal tissue (1), including the prevention of osteoradionecrosis after dental extraction, the treatment of mandibular osteoradionecrosis in combination with surgery and the treatment of haemorrhagic cystitis resistant to conventional treatments. There are several studies published on HBO therapy for radiation cystitis, but most of them have discrepancies regarding their design and methodology since they are retrospective and not randomized or comparative; they lack control group and HBO is used as a secondary treatment. Herein, we present our updated results of a study, whose initial results concerning patients recruited until 2010 have been already published (2). Our present study includes the first and sole prospective series on HBO therapy of severe radiation cystitis in patients who have not received any previous treatment.

## MATERIALS AND METHODS

### Inclusion/Exclusion criteria

Since September 2007 we have prospectively enrolled 38 patients with severe radiation cystitis (initially grade IV post-radiation haematuria, according to the RTOG and the EORTC acute and late radiation morbidity scoring criteria for radiation-induced haemorrhagic cystitis) (Table-1) (3). All patients enrolled suffered from severe haemorrhagic cystitis with haematuria requiring transfusion. All patients should have only had a urethral catheter and bladder irrigation as initial and unique treatment of their radiation-induced haemorrhagic cystitis and may have started transfusions prior to HBO therapy. Patients with severe emphysema or other severe chronic obstructed airway disease, a history of spontaneous pneumothorax, of tympanic membrane spontaneous perforation or otological reconstruction were excluded from our study, as well as patients with active viral infection, history of treatment with cisplatin or doxorubicin, active bladder malignancy and uncorrected bleeding disorders.

**Table 1:** Classification of haematuria events for both acute and late radiation morbidity scoring criteria for radiation-induced haemorrhagic cystitis ( 3 ).

	Radiation morbidity	
Haematuria morbidity	Acute* (RTOG**)	Late* (RTOG/EORTC***)
Grade I	NA	Minor telangiectasia (microscopic haematuria)
Grade II	NA	Generalized telangiectasia (macroscopic haematuria)
Grade III	Gross haematuria with or without clot passage	Severe generalized telangiectasia (frequent macroscopic haematuria)
Grade IV	Haematuria requiring transfusion	Severe haemorrhagic cystitis
Grade V	Death from uncontrolled haematuria	Death from uncontrolled haematuria

s*Acute morbidity defined as treatment related complications occurring within 90 days from first radiotherapy session

**RTOG: Radiation Therapy Oncology Group

***EORTC: European Organization for Research and Treatment of Cancer

### Evaluation and Treatment

On admission to hospital all patients underwent the standard laboratory examinations (2) including full blood count and complete clotting and biochemistry profile measurements. Urine samples were examined for common urinalysis, urine culture and cytology. Computed tomography (CT) or Magnetic Resonance Imaging (MRI) of the abdomen was scheduled for staging purposes and to exclude other bladder pathology. All patients underwent cystoscopy under anesthesia and bladder biopsies were taken by one surgeon (A.D) to confirm histological changes consistent with radiation cystitis and to exclude bladder malignancy. A large diameter urethral catheter (22F-24F) was placed to secure prompt bladder irrigation.

All patients were initially scheduled to receive 30HBO sessions in a walk-in multi-place hyberbaric chamber with intend to increase them up to 45 sessions until the haematuria resolved. According to the routine protocol of our department, all patients are planned to receive 100% oxygen at a 1.8 atmospheres absolute pressure per session for 90 minutes per day, five days a week - Monday to Friday. When complete response to HBO was reached the treatment was ceased. In case of worsening or relapse of haematuria during follow-up, HBO therapy was re-initiated under the same schedule. In case no benefit was gained from the initial treatment, more than 45 treatments were needed, severe complications occurred, or if patients declined further therapy, HBO was considered as a failure and patients were referred for further treatment (conservative or surgical).

Within 4 weeks of treatment completion, all patients underwent cystoscopy under anesthesia by the same surgeon to confirm treatment result and/or to compare with the pre-treatment status. Bladder biopsy was performed in only 19 initial patients and was further abandoned, because pathology reports were stable and we considered we should therefore avoid any morbidity. This procedure was further reserved only for patients with subjectively abnormal bladder mucosa.

### Study endpoints

Primary endpoint was the success rate measured by the incidence of complete and partial response to treatment. Complete response was defined as the complete cessation of bleeding and the lack of need for transfusion in combination with the disappearance of endoscopic findings and concomitant normal bladder findings in repeat biopsies where available. Partial response was defined as a decrease in the grade of RTOG/EORTC scoring criteria, the existence of microscopic haematuria or the persistence of mild macroscopic haematuria not requiring transfusion or other urgent treatment. Secondary endpoints include the duration of HBO response without the need for further treatment, the number of sessions needed to achieve success, the avoidance of surgery and the overall survival.

### Statistical analysis

Descriptive statistics and comparisons were made using the SPSS 16 (SPSS Inc. Chicago IL) statistical package with p<0.05 being significant. Statistical analysis was performed using the chi-square and t test, as appropriate.

## RESULTS

Since September 2007 thirty-eight patients (thirty-three men and five women) were enrolled in our study. Mean patient age was 70.3 years (range 56 to 82). Indications for radiation therapy included prostate cancer for twenty-eight, muscle-invasive bladder cancer for seven, rectal cancer for one and cervical cancer for two patients. Mean radiation dose was 63.8Gys (range 32 to 80), while there were no data available in 3 patients. Mean interval between completion of radiation therapy and onset of haematuria was 21.4 months (range 1 to 210). Mean interval between completion of radiation therapy and the onset of HBO therapy was 24.7 months (range 2 to 212 months). Mean time interval between the onset of the hematuria to the HBO treatment was 5.4 months (range 1-48). Mean transfusion need prior to and during the treatment was 7.6 red blood cells packs (range 3 to 16). In particular, 11 patients were transfused with up to 6 units and 27 patients with more than 6 units.

Pre-treatment patient evaluation revealed no bladder infection or tumour in any case. Pathology reports were stable and included histological findings consistent with post-radiation cystitis in all cases; that were: diffuse mucosal edema, vascular telangiectasia, submucosal haemorrhage and interstitial or smooth muscle fibrosis. Severe ischemia of the bladder wall as a result of obliterative endarteritis, was also occasionally present.

All patients completed HBO therapy without experiencing any severe HBO-related complications and were followed-up for a mean period of 29.3 months (range 3 to 94). Mean HBO therapy sessions were 33 (range 20 to 78). Thirty-three patients (86.8%) had complete response while five patients (13.2%) experienced partial response with marked improvement in their haematuria (grade II). For the thirty-three patients with complete response who received HBO therapy within 6 months of the haematuria onset, the mean time interval was 4.9 months (range 1-6), while in the remaining five patients with partial response the mean time interval was 22 months (range 8-48) (p<0.001). All demographics and results of our study are detailed in Table-2. One patient from the complete response group had a recurrence of grade II haematuria at 6 months of follow-up and received 18 additional HBO treatments. All aforementioned patients with complete response remained stable for the rest of the follow-up. Three patients with partial response received 15 additional treatments and they had not had haematuria since then. The two last patients experienced severe haematuria 6 months after the end of HBO therapy and following a full consent one underwent cystectomy and urinary diversion, while the other was offered a successful embolisation with sporadic episodes of low severity macroscopic haematuria since then.

**Table 2:** Demographics and results of our study on hyperbaric therapy in the treatment of radiation-induced bladder complications.

	n
Patients	38
Men	33
Women	5
Age (years)	70.3 (56-82)*
**Indications**	
Prostate Cancer	28
Muscle-Invasive Bladder Cancer	7
Rectal Cancer	1
Cervical Cancer	2
Radiation Dose (Gys)	63.8 (32-80)*
Not Available Data	3
Interval Between haematuria to HBO (months)	5.4 (1-48)*
Interval between Radiotherapy to haematuria (months)	21.4 (1-210)*
Interval between radiotherapy to HBO (months)	24.7 (2-212)*
Transfusion	7.6 (3-16)*
≤6 units	11
>6 units	27
Follow-up (months)	29.3 (3-94)*
HBO sessions	33 (20-78)*
**Response**	
Complete	33 (86.8%)
Partial	5 (13.2%)

*mean (range)

Post-hyperbaric treatment cystoscopy revealed a subjectively normal bladder mucosa in thirty two patients, which was confirmed by a histologically normal mucosa in 17 of the patients who had complete response. The embolised patient showed improved but persistent findings of radiation cystitis. The pathologic examination of the cystectomy specimen revealed, apart from findings of radiation cystitis, a transitional T2G3 muscle invasive bladder cancer (MIBC).

Regarding our study endpoints, complete response rate was 86.8% and partial response rate was 13.2%, giving an overall success rate of primary therapy of 100%. It should be underlined that all 33 patients with complete response received HBO therapy within 6 months of the haematuria onset compared to the remaining 5 patients with partial response who received HBO therapy ranging from 8 to 48 months from the haematuria onset. Thirty-three patients were alive at the end of follow-up.

## DISCUSSION

Pelvic radiation may result in either acute or chronic bladder injuries (4) that lead to radiation-induced haemorrhagic cystitis, in 5-10% of cases (5). Bladder complications may occur within two months to more than twenty years following completion of pelvic radiotherapy (1, 6) with haemorrhage being present in 9% of cases (1).

Severe RTOG/EORCT grade III or worse bladder morbidity has been reported at 1% at 5 years, 1.4% at 10 years and 2.3% at 20 years following radiotherapy for cervical cancer (7). Radiation for prostate cancer may lead to moderate or severe haematuria in 3-5% of cases (8).

Traditionally, severe radiation cystitis has been treated in various ways (1). Continuous or intermittent bladder irrigation with large bore catheters usually constitutes the first-line treatment. Intravesical instillations with alum, silver nitrate, phenol, formalin or hyaluronic acid have been used as a second-line treatment, while the third-line therapy is consisted of several oral and intravenous agents, such as aminocaproic acid, traxenamic acid, corticosteroids, estrogens, antibiotics, prostaglandins and sodium pentosanpolysulphate. These agents are administered either concomitantly with first or second-line treatment options or as a pure third-line treatment. Unfortunately, traditional treatments are not well validated in terms of efficacy and constitute results from non-randomized trials in most of the cases grossly underpowered. Furthermore, there are no prospective studies comparing oral, intravesical and intravenous treatments between them or with HBO, apart from one randomized study between intravesical hyaluronic acid instillation and HBO therapy with similar results (9). However, all these treatments do not cure the radiation-induced cystitis, nor prevent recurrence of severe haematuria. Moreover, some of them may have serious systematic side effects or may exacerbate bladder fibrosis which was initiated by radiation treatment, leading to a small-capacity low-compliant urinary bladder (1, 8, 10-17). More than a decade ago, a Cochrane Database systematic literature review on non-surgical interventions for late radiation cystitis in patients who underwent radical radiotherapy to the pelvis concluded that in the absence of randomized controlled studies it is impossible to set definitive rules for treatment (5). Recently, there have been reports of KTP laser use in an attempt of haemostasis with minimal mucosal destruction with promising results as a final step before definitive treatment (18, 19). In case of intractable haemorrhage, arterial embolization or ligation and/or cystectomy represent definitive treatment, at the cost of increased morbidity.

Urothelial cellular changes due to radiotherapy are caused by water radiolysis. This results in increase of activated free oxygen radicals that cause cell-membrane injury by lipid peroxidation and immediate cell death. Furthermore, DNA damage caused directly by radiation energy per se and indirectly by free oxygen radicals results in replication failures and further cell death (20). Additionally, pelvic radiotherapy initially causes mucosal edema and inflammation. Telangiectasia, submucosal haemorrhage and interstitial fibrosis may follow. Obliterative endarteritis of small blood vessels leads to acute and chronic ischemia of the bladder wall and eventually to smooth muscle fibrosis due to cellular hypoxia. The later chronic endarteritis is successfully described as “the three-H model” (21, 22).

Hyperbaria related to HBO therapy increases bladder’s tissue oxygen tension (17). Hyperoxia enhances neovascularization and growth of normal tissue (8, 23). Angiogenesis is stimulated by tissue macrophages responding to the steep oxygen gradient. Interestingly, the tissue oxygen remains almost in normal levels for many years following HBO therapy, implying that the hyperoxia-induced angiogenesis is essentially permanent (8).

Vasoconstriction and cease of bleeding as well as improvements of tissue healing and immune function constitute additional beneficial effects of HBO (23).

The use of HBO should not be thought as a treatment modality that cures everything although there are cases where irrelevant improvements to HBO therapy have been reported (24). Especially, as far as radiation-induced haemorrhagic cystitis is concerned, several series of patients treated with HBO have been published (6, 8, 10-17, 21, 25-30). The vast majority are retrospective reviews and case series with only a few ones (6, 17, 26, present study) being prospective in nature. Furthermore, a recent review raised concerns on whether HBO therapy shows clear clinical benefit on radiation cystitis (25). In all these studies HBO was used as a secondary treatment option. In experimental setting (23) HBO may correct the underlying pathophysiology of radiocystitis, leading to permanent cure.

To the best of our knowledge, our study is the first and sole prospective study on only severe haematuria patients. It is the first study using HBO as primary therapy for radiation-induced cystitis and the first in which post-treatment cystoscopic and histologic findings were included as study’s endpoints. Furthermore, apart from the absolute overall success rate of HBO as primary therapy, complete response rate in 86.8% of cases is the highest in literature. The highest efficacy of our suggested method is further amplified by the fact that patients are stable with no or minor radiotherapy-induced morbidity for a relatively long follow-up period. As a result, we can therefore conclude that primary treatment of severe post-radiation haemorrhagic cystitis with HBO has proved to be effective and safe both for the bladder structure itself and for patients and should be underlined that none of our patients had to discontinue HBO therapy due to HBO side effects. Initiation of therapy within 6-months of haematuria onset seems to be of utmost benefit, since in this early setting HBO therapy is assumed to break the vicious circle of chronic sloughing and resultant scarring in cases of hypoxic irradiated bladder tissues (16). Finally, we support the findings of other series (10, 11), indicating that when HBO fails, the urologist should consider other underlying causes such as malignancy.

Our study has several drawbacks. First of all, it is not randomized or controlled, but given the fact that cystectomy represents the alternative definitive treatment for radiation cystitis we believe it will be difficult to randomize patients. Secondly, due to strict inclusion criteria, since it is the only prospective study with severe haemorrhagic cystitis patients all of which needed transfusion, the number of patients enrolled in our study is relatively small.

## CONCLUSIONS

The early primary use of hyperbaric oxygen to treat severe radiation-induced haematuria (especially within the first six months from the haematuria onset) is an effective and safe treatment option with excellent initial results. Increasing patient recruitment, precise and longer follow-up is warranted to extract careful and permanent conclusions. Prospective randomized controlled trials uniformly designed in order to avoid variability in treatment strategies, will eventually provide more precise information.
